# Electrochemical stability and neural recording with sputtered ruthenium oxide electrodes subchronically in rat motor cortex

**DOI:** 10.1088/1741-2552/adee49

**Published:** 2025-07-21

**Authors:** Justin R Abbott, Eleanor N Jeakle, Yupeng Wu, Thomas J Smith, Negar Geramifard, Bitan Chakraborty, Mahasty Khajehzadeh, Sahana Dhananjayan, Ana G Hernandez-Reynoso, Joseph J Pancrazio, Stuart F Cogan

**Affiliations:** 1 Department of Bioengineering, The University of Texas at Dallas, Richardson, TX, United States of America; 2 Department of Materials Science and Engineering, The University of Texas at Dallas, Richardson, TX, United States of America; 3 School of Behavioral and Brain Sciences, The University of Texas at Dallas, Richardson, TX, United States of America

**Keywords:** ruthenium oxide, neural recording, microelectrodes, amorphous SiC, thin-films

## Abstract

*Objective*. To investigate sputtered ruthenium oxide (RuO*_x_*) as a low impedance coating for recording neural activity with intracortical microelectrode arrays (MEAs). *Approach.* RuO*_x_* was sputtered onto active sites of amorphous silicon carbide (a-SiC) MEAs and evaluated electrochemically and as a recording electrode coating over 6 weeks in rat motor cortex. The RuO*_x_* was 250 nm thick with a 200 *μ*m^2^ geometric electrode surface area. We performed weekly electrochemical impedance spectroscopy and cyclic voltammetry (CV) measurements and single-unit action potential recordings. *Main results.* From 1 to 6 weeks post-implantation, we observed that median 1 kHz impedance decreased from 1.06 MΩ to 0.68 MΩ, median 1 Hz impedance decreased from 24.5 MΩ to 20.7 MΩ, and median 30 kHz impedance remained constant at 0.19 MΩ. Linear regression fits indicated that the 1 kHz and 1 Hz impedance exhibited small decreases whereas the 30 kHz impedance values had no discernible trend. The CV-based charge storage capacity (CSC_c_) also exhibited consistency over time. The median CSC_c_ at 50 mV s^−1^ was 24.3 mC cm^−2^ on week 1 and 20.1 mC cm^−2^ at week 6. At 50 000 mV s^−1^ sweep rate, the median CSC_c_ was 2.4 mC cm^−2^ at 1 week and 2.8 mC cm^−2^ at 6 weeks. Regressions for both sweep rates exhibited no significant slope deviations from zero. Neural recordings spanning 6 weeks demonstrated consistent single unit action potential with a 75% single unit active-electrode-yield over 6 weeks. Median *V*_pp_ values on week 1 were 97.6 *µ*V and 105 *µ*V by week 6. The median and quartile signal-to-noise ratio (SNR) was 12 at week 1 and 12 at week 6. There were no deviations from zero on the regressions for both *V*_pp_ and SNR. *Significance.* The findings suggest that RuO*_x_* is stable over subchronic implantation periods. Future research will explore long-term chronic performance and broader applications of RuO*_x_* in neural interface devices.

## 1. Introduction

Cortically implanted microelectrode arrays (MEAs) are a critical part of brain machine interfaces that connect devices, such as sensory or motor prostheses, to the brain and provide insight into the functional circuitry comprising complex neuronal networks [1–4]. A key component of these devices is the electrode material that transduces the ionic flow underlying neural signals to electronic potentials at the recording electrode. These electrode materials must have a low enough impedance to record extracellular action potentials, demonstrate stability in the cortical environment, and not induce adverse reactions that might damage cortical neurons. There has been success using capacitive films, such as titanium nitride, or faradaic films, such as iridium oxide or poly(ethylenedioxythophene), to serve as the recording interface [5–9]. Recently, the use of water vapor in the reactive sputtering of sputtered iridium oxide film (SIROF) has been shown to increase the charge storage and injection capacities and reduce the impedance of SIROF electrodes without increasing their geometric size [10]. The decrease in the impedance facilitates the fabrication of smaller electrode sites without compromising recording capabilities [11, 12]. These SIROF coatings have demonstrated stable neural recordings in a chronic implantation in rat motor cortex [13]. Additionally, multielectrode arrays fabricated from amorphous silicon carbide (a-SiC) using SIROF as a low impedance electrode coating have been described [13–15]. Recently, sputtered ruthenium oxide (RuO*_x_*) was investigated as a potential film for neural interface electrodes [16, 17]. Thin films of sputtered RuO*_x_* were found to have similar charge injection and impedance properties to those of SIROF, as well as a similar faradaic process for storing and delivering charge [17]. RuO*_x_* has also been investigated for use in cardiac pacemakers [18]. At the time of writing, iridium is about seven times more expensive than ruthenium. Due to its lower cost, amenability to thin film fabrication techniques for neural interfaces, and similar impedance and charge injection capabilities, RuO*_x_* represents a promising alternative to the more expensive iridium options currently in use. Prior work has demonstrated that RuOx has the capacity to record neural signals acutely in rat motor cortex [16]. Here we extend the study to a 6 week, short chronic implantation in rat motor cortex. To provide a direct comparison, we assessed the stability of the RuO*_x_* recording electrodes following the methodology reported previously for SIROF coatings on identical a-SiC MEAs [13]. These assessments encompassed *in vivo* electrochemical impedance spectroscopy (EIS) and cyclic voltammetry (CV) as well as weekly neural recordings to monitor changes in single unit extracellular action potentials. The neural recordings were quantified by the number of single units identified, unit amplitudes and signal-to-noise ratio (SNR) to assess changes in recording capability over the 6 week implantation period. Overall, our findings demonstrate that RuO*_x_* electrode coatings maintain electrochemical and recording stability over a subchronic period of 6 weeks in rat cortex.

## 2. Materials and methods

### 2.1. Electrode array fabrication

**Figure 1.**
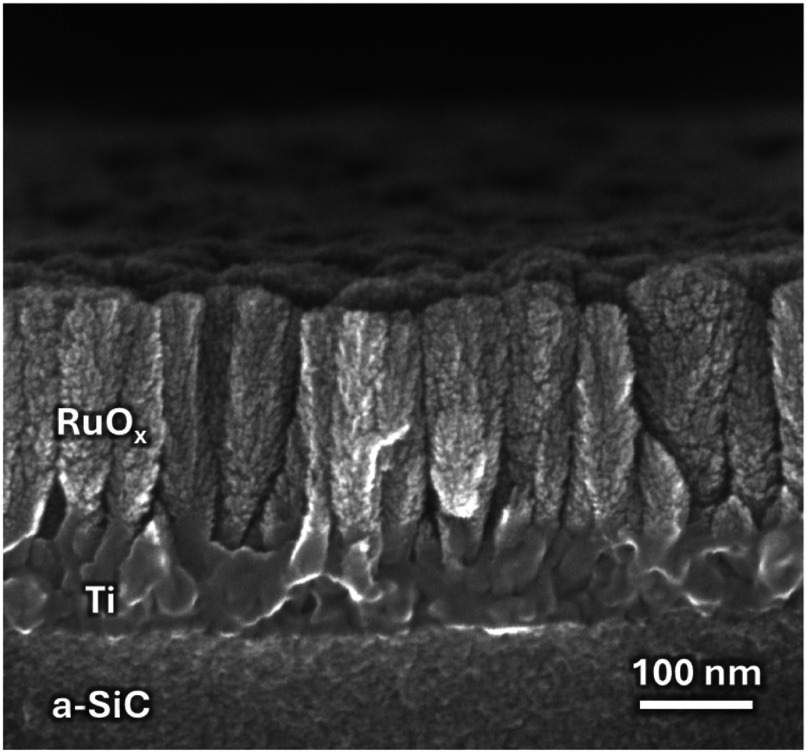
SEM image of the cross-sectional structure of sputtered ruthenium oxide (RuO*_x_*) film. The cross-section was obtained by cleaving a monitor wafer coated in a-SiC. The a-SiC base layer, Ti adhesion layer, and RuO*_x_* film layers are labeled. The RuO_x_ shown is approximately 200 nm thick, slightly thicker than the implanted 120 nm films, and the Ti adhesion layer approximately 50 nm thick.

**Figure 2.**
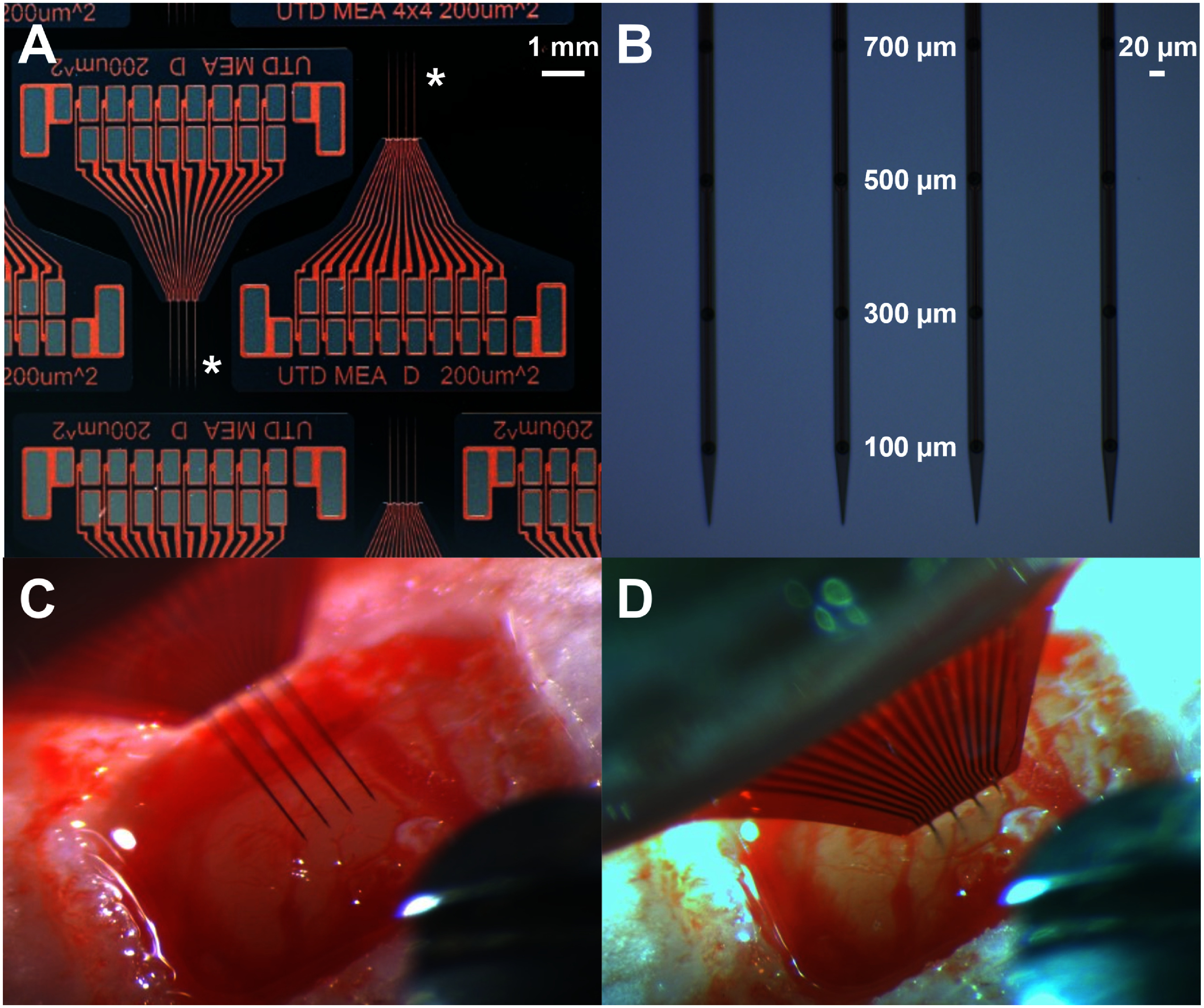
(A) Optical image of four shank a-SiC devices with RuO*_x_* electrode sites on wafer following fabrication. Penetrating shanks are marked with a * in A. (B) Image of the shanks and RuO*_x_* recording electrode sites along the shank. The electrode sites are connected to the bond pad region by an etched via through the top layer of a-SiC to make electrical contact with the underlying gold metallization layer. Markers for distance between tip of the shank and center of the electrode site can be seen between the two inner shanks in B. Before (C) and after (D) implantation of an array into rat motor cortex. The size of the craniotomy shown is approximately 3 × 2 mm.

MEAs of a-SiC were fabricated at the cleanroom facility at The University of Texas at Dallas using the fabrication process described in [13]. A cross-sectional SEM of the porous structure of RuO*_x_* is shown in figure 1. Devices were fabricated to have 4 collinear penetrating shanks. Each shank was 20 *µ*m wide by 8 *µ*m thick and with a 2 mm length. Four 200 *µ*m^2^ surface area RuO*_x_* disk recording electrode sites per shank were patterned and deposited using reactive DC magnetron sputtering as described in [17]. The measured thickness of the RuO_x_ films was approximately 120 nm. Additionally, a 50 nm Ti adhesion layer was deposited before the RuO*_x_*. The center of each electrode is located at 100, 300, 500, and 700 *µ*m from the tip of each penetrating shank. Once device fabrication was completed, devices were soaked in room temperature deionized water for 72 h to hydrate a thin 500 nm polyimide base-layer to release the devices from the wafer. Devices were then bonded to connectors (Omnetics Connector Corporation, USA) using conductive silver paste (Sigma-Aldrich, St. Louis, MO, USA). 30G stainless-steel reference and ground wires were also attached using the same silver paste. The bond pad area was coated in an insulating material (EA M-121HP, Henkel Loctite, Germany) to encapsulate the device external to the brain parenchyma. Images of completed devices are shown in figures 2(A) and (B).

### 2.2. Surgical implantation

The University of Texas at Dallas Institutional Animal Care and Use Committee (protocol #18-13) granted approval for all procedures conducted in this study. Seven adult female Sprague Dawley rats were used. Female rats were chosen to compare to previous work that investigated SIROF recording electrodes [13]. To initiate anesthesia, 3% isoflurane in oxygen was administered. A surgical plane of anesthesia was assured by performing tail and toe pinches. Subsequently, animals were placed in a stereotactic frame. Anesthesia was maintained with 2% isoflurane in oxygen. The cortex was accessed by a midline incision on the scalp and tissue was removed to expose the cranium. Stainless steel bone screws were inserted adjacent to the implantation site. A craniotomy of approximately 3 × 2 mm in dimension was made. The dura was removed over the left motor cortex (M1) at 2 mm anterior from bregma and 2 mm lateral from the midline. Ground and reference wires were secured around the bone screws. A NeuralGlider Cortical Neural Implant Inserter (Actuated Medical, Inc., USA) was used to facilitate implantation by providing axial vibration along the insertion path. The arrays were inserted at a speed of 100 *µ*m s^−1^ to a depth of approximately 1.5 mm with a vibrational power of 0.5 W. Images before and after implantation can be seen in figures 2(C) and (D), respectively. Following implantation, a collagen dural graft replaced the removed dura and a topical adhesive was used to cover the craniotomy. Finally, dental cement was used to create a headcap that fully encapsulated the external device, bone screws, and exposed cranium.

### 2.3. Electrochemical evaluation

RuO_x_ electrode stability was assessed by performing two electrochemical measurements, EIS and CV, on each channel of the implanted devices throughout the 6 week implantation period. EIS measurements encompassed a frequency range of 1–10^5^ Hz, at 10 points per decade, applying a 10 mV root mean square (RMS) (centered around the open circuit potential measured with a Ag|AgCl reference electrode) sinusoidal voltage signal to each RuO*_x_* electrode. Impedance magnitude was investigated at three frequencies: 1 Hz, 1 kHz, and 30 kHz. These frequencies were selected due to their association with local field potentials, single unit action potentials, and tissue impedance [19, 20]. CV measurements involved cycling each RuO*_x_* electrode over a potential range of +0.6 V to −0.6 V versus Ag|AgCl at 50 mV s^−1^ and 50 000 mV s^−1^ sweep rates. These measurements were conducted to monitor the functionality of the electrode and to observe faradaic reactions occurring at the electrode site. The +0.6 V to −0.6 V potential range is a conservative estimate of the water electrolysis limits previously identified for sputtered RuO*_x_* [17]. The cathodal current over a single CV cycle was integrated over time to determine the cathodal charge storage capacity (CSC_c_). Both *in vivo* and pre-implantation saline EIS and CV measurements were conducted using a Gamry Reference 600 potentiostat (Gamry Instruments Inc, Warminster, PA, USA). All electrochemical measurements were performed in a 3-electrode configuration.

The pre-implantation CV and EIS measurements were made in an inorganic model of interstitial fluid (mISF) saturated with a CO_2_/O_2_/N_2_ gas mixture (5/6/89%, respectively). The mISF measurements employed a large-area Pt wire counter-electrode and a Ag|AgCl reference electrode (BASi Research Products, West Lafayette, IN, USA). The CV and EIS measurements in mISF served to confirm the functionality of each electrode channel and to provide baseline data for comparison with *in vivo* measurements. For the *in vivo* measurements, a stainless-steel needle inserted intradermally into the rat’s tail served as the counter-electrode and a chlorodized silver pad, electrically connected to the tail with conductive gel, served as a reference electrode. The *in vivo* electrochemical measurements were initiated 7–13 d after the implantation and were conducted weekly thereafter over a 6 week implantation period. During electrochemical measurements, rats were anesthetized using 3% inhaled isoflurane and anesthesia was maintained with 1.5% isoflurane.

CV measurements were used to assess whether an electrode site remained functional or if there was a disconnection between the site and the connector channel output. If the CSC_c_ for a specific channel, determined by the 50 mV s^−1^ sweep CV, fell below 1 mC cm^−2^ and the CSC_c_ from the 50 000 mV s^−1^ sweep was below 0.1 mC cm^−2^, we inferred that the channel had become disconnected. Such CVs, characterized abnormally low CSC_c_ values, were indicative of a lack of observable oxidation-reduction peaks associated with ruthenium oxide. Disconnected channels were not included in the analysis for electrochemistry or neural recordings. In rare instances, a channel might exhibit a disconnected status for one week but then appear to be reconnected in subsequent weeks. We hypothesize that this is primarily due an intermittent contact at the Omnetics connector, either at connections to the a-SiC MEA or between mating halves of the connector. In such cases, data from the week with observed electrochemical disconnects were excluded from electrochemical analysis. However, given the intermittent contact and since electrochemical measurements and neural recording used different connectors, these channels were considered connected when calculating the active electrode yield (AEY) for neural recording assessments for that week. Indeed, there are instances in which a channel may appear disconnected electrochemically and able to record presumptive single units on the same week. In these cases, electrochemical connectivity returned the following week. The intended total number of fabricated electrode channels was 112 (16 channels for each of 7 devices) devices). As identified by electrochemistry, 112 electrodes were available for recording prior to implantation and all 112 were functional 1 week post-implantation. At the 6 week endpoint, 92 channels were functional. Based on the characteristic open circuit EIS and CV responses, the primary cause of functionality loss is likely failure of interconnects external to the brain parenchyma.

### 2.4. Neural recordings

Immediately after electrochemical measurements each week, wideband neural recordings spanning 0.1–7000 Hz were conducted on anesthetized rats (1.5% inhaled isoflurane) for 600 s. Neural recordings were acquired at a sampling frequency of 40 kHz using a Blackrock Cereplex system (Salt Lake City, USA). The raw recording data were bandpass filtered from 300 Hz to 3000 Hz using a 4-pole Butterworth filter, with common median referencing applied to reduce noise and minimize artifacts [21]. Single-unit identification was achieved by applying a −4*σ* (standard deviation) threshold to the filtered signal, followed by an automated k-means scan and manual verification based on unit separation in principal component space. The recording performance of the RuO*_x_* devices was evaluated by calculating the AEY, representing the percentage of functional channels successfully recording at least one single unit per week. SNR was assessed by dividing the peak-to-peak voltage (*V*_pp_) of isolated single units by the baseline noise level. The noise level was determined by removing segments of the filtered continuous signal associated with single units from the overall signal and then calculating the RMS of the remaining signal. Electrode channels identified as disconnected through electrochemical analysis were excluded from the recording performance analysis.

### 2.5. Statistical methods

Normality was evaluated using the Shapiro–Wilk’s test. All electrochemical datasets were non-normally distributed, exhibiting a positive skewness toward larger values in both impedance and CSC_c_ values. Electrochemical data are represented by medians, quartiles, and ranges. Outliers were identified and removed from the dataset if they exceeded three times the interquartile range of the data. To compare mISF and *in vivo* measurements, Mann–Whitney *U* Tests were conducted for each electrochemical measurement across the two datasets. The influence of post-implantation time was examined using on the *in vivo* time series data, with deviation from zero in the resulting slopes and the 95% confidence interval (CI) of the slope reported. Statistical significance was set at *p* < 0.05. Similarly, neural recording data, which were non-normally distributed, were analyzed using the same approach as the electrochemical data. Statistical analyses were performed using Stata (College Station, TX, USA) and GraphPad Prism (Dotmatics, Boston, MA, USA).

## 3. Results

### 3.1. Electrochemical evaluation

**Figure 3.**
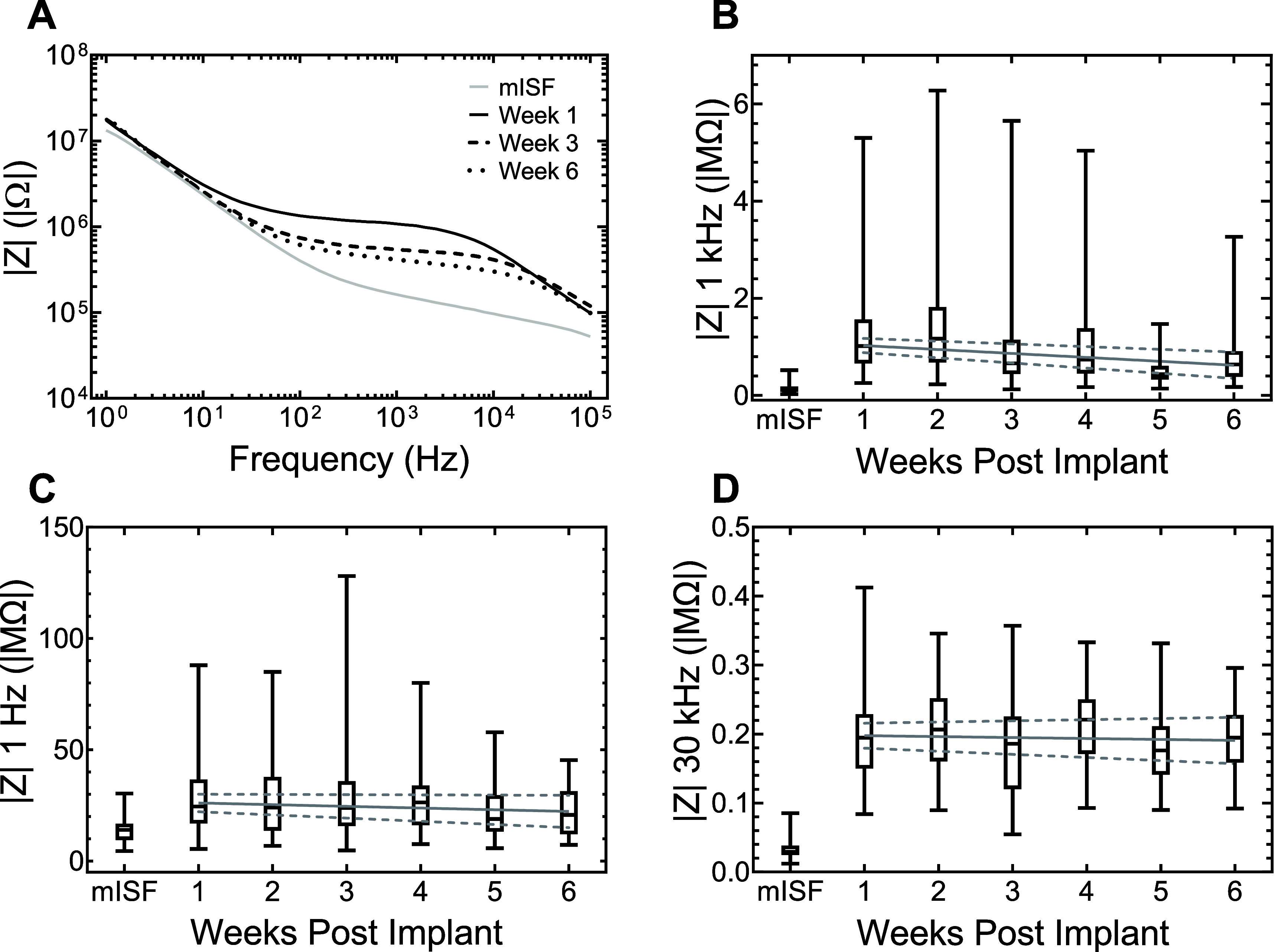
(A) Representative EIS bode plots in mISF and over the 6 week implantation period from a single electrode; (B) median1 kHz, quartile, and range values over all animals; (C) median 1 Hz, quartile, and range values over all animals; (D) median 30 kHz, quartile, and range values over all animals. Robust linear regression trendlines with a 95% confidence interval (dashed lines) are plotted on B, C, and D.

EIS spectra and impedance over the 6 week implantation period are shown in figure 3. Figure 3(A) shows changes in the EIS spectra for a representative electrode in mISF and at 1-, 3-, and 6 weeks post-implantation. Figures 3(B)–(D) show weekly medians, as well as quartiles and ranges, of impedance magnitude at frequencies of 1 kHz, 1 Hz, and 30 kHz, respectively, averaged over all animals. The pre-implantation median values of 0.097 MΩ (1st quartile: 0.073 MΩ, 3rd quartile: 0.18 MΩ) at 1 kHz, 14.0 MΩ (9.52 MΩ, 16.7 MΩ) at 1 Hz, and 0.029 MΩ (0.024 MΩ, 0.038 MΩ) at 30 kHz. Following implantation, the median 1 kHz impedance increased to 1.06 MΩ (0.67 MΩ, 1.54 MΩ). The 1 Hz and 30 kHz impedance values also increased to 24.5 MΩ (16.9 MΩ, 36.5 MΩ) and 0.19 MΩ (0.15 MΩ, 0.22 MΩ), respectively. The increases in impedance magnitudes from mISF to cortex were significant at all three frequencies analyzed (*p* < 0.001, Mann–Whitney *U* Test). Over the 6 week *in vivo* period, the median 1 kHz impedance decreased from 1.06 MΩ to 0.63 MΩ (0.39 MΩ, 0.91 MΩ). The median impedance at 1 Hz also declined slightly from 24.5 MΩ to 20.7 MΩ (12.1 MΩ, 30.9 MΩ), and median 30 kHz remained stable at 0.19 MΩ (0.16 MΩ, 0.22 MΩ) at week 6. Post-implantation impedance declined from week 1 to week 6 most notably in the mid-frequency range, as seen in figure 3(A). For the 1 kHz data, a robust linear regression revealed a slight negative trend (*p* < 0.001, slope = −0.081 MΩ week^−1^, 95% CI = (−0.056 MΩ week^−1^, −0.11 MΩ week^−1^)). Linear regression analysis also revealed a small, but significant, negative trend in 1 Hz impedance over 6 weeks (*p* = 0.025, slope = −0.76 MΩ week^−1^, 95% CI = (−0.97 MΩ week^−1^, −1.4 MΩ week^−1^)). No trend was observed in the 30 kHz impedance (*p* = 0.389, slope = −0.0013 MΩ week^−1^, 95% CI = (−0.0045 MΩ week^−1^, 0.0017 MΩ week^−1^)). Despite significant trendlines at 1 kHz, the small slope magnitudes suggest overall stability of the RuO*_x_* film *in vivo* over the 6 week period.

**Figure 4.**
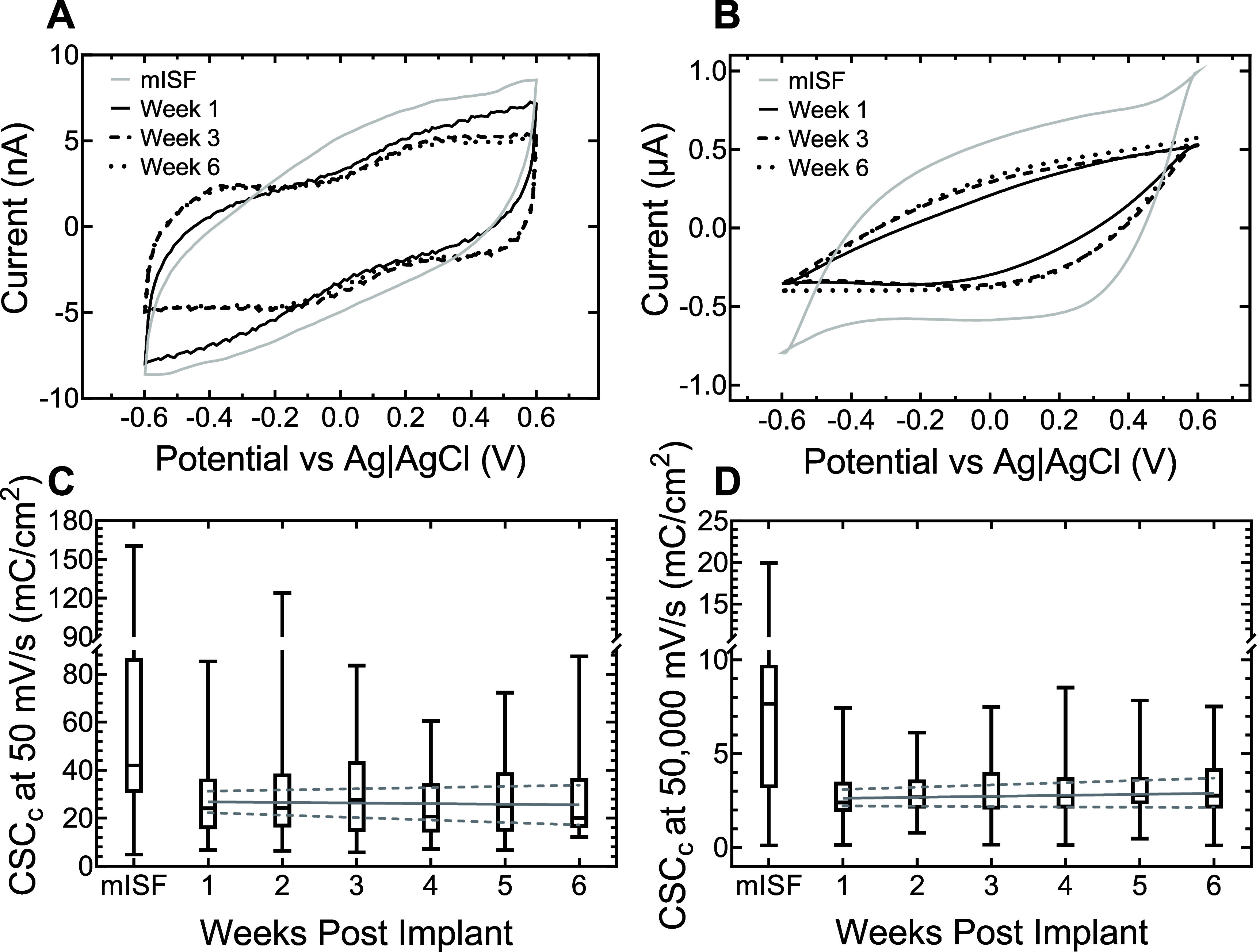
Representative CV curves at (A) 50 mV s^−1^ and (B) 50 000 mV s^−1^ sweep rates for a RuO*_x_* electrode site in mISF and at weeks 1-, 3, and 6 post-implantation. Box and whisker plots represent the median, quartiles, and overall range for CSC_c_ at (C) 50 mV s^−1^ and (D) 50 000 mV s^−1^. These data depict the CSC_c_ of all available electrodes over all arrays each week. Robust linear regression trendlines with a 95% confidence interval (dashed lines) are plotted on C and D.

As expected, CV measurements and calculated CSC_c_ at 50 mV s^−1^ and 50 000 mV s^−1^ exhibited a significant decrease between mISF and rat cortex. Representative 50 mV s^−1^ and 50 000 mV s^−1^ sweep rate CV curves from a single RuO*_x_* electrode are shown in figures 4(A) and (B), respectively. Figures 4(C) and (D) show the median CSC_c_, accompanied by quartiles and range values as well as a robust regression fit, for each sweep rate over 6 weeks. In mISF, median and quartile CSC_c_ values were 41.9 mC cm^−2^ (30.6 mC cm^−2^, 85.3 mC cm^−2^) at 50 mV s^−1^ and 7.7 mC cm^−2^ (3.2 mC cm^−2^, 9.7 mC cm^−2^) at 50 000 mV s^−1^. In the first week following implantation, median CSC_c_ decreased to 24.3 mC cm^−2^ (16.7 mC cm^−2^, 36.4 mC cm^−2^) at 50 mV s^−1^. By the sixth week post-implantation, the median 50 mV s^−1^ CSC_c_ measured 20.1 mC cm^−2^ (16.1 mC cm^−2^, 35.9 mC cm^−2^). The mISF and week one 50 mV s^−1^ CSC_c_ exhibited a significant change (*p* < 0.001, Mann–Whitney *U* Test). A robust linear regression applied to the *in vivo* CSC_c_ data indicated no significant trend in 50 mV s^−1^ CSC_c_ over 6 weeks (*p* = 0.494, slope = −0.264 mC cm^−2^ week, 95% CI = (−1.02 mC cm^−2^ week, 0.492 mC cm^−2^ week). The 50 000 mV s^−1^ CSC_c_ declined to 2.40 mC cm^−2^ at 1 week after implantation (1.91 mC cm^−2^, 3.47 mC cm^−2^), showing a significant difference (*p* < .0001, Mann–Whitney *U* Test). The median 50 000 mV s^−1^ CSC_c_ remained fairly constant over 6 weeks, reaching 2.76 mC cm^−2^ (2.10 mC cm^−2^, 4.19 mC cm^−2^) by week six. Robust linear regression indicated no significant trend in the 50 000 mV s^−1^ sweep CSC_c_ (*p* = 0.137, slope = 0.053 mC cm^−2^ week, 95% CI = (−0.017 mC cm^−2^ week, 0.12 mC cm^−2^ week).

### 3.2. Neural recordings

**Figure 5.**
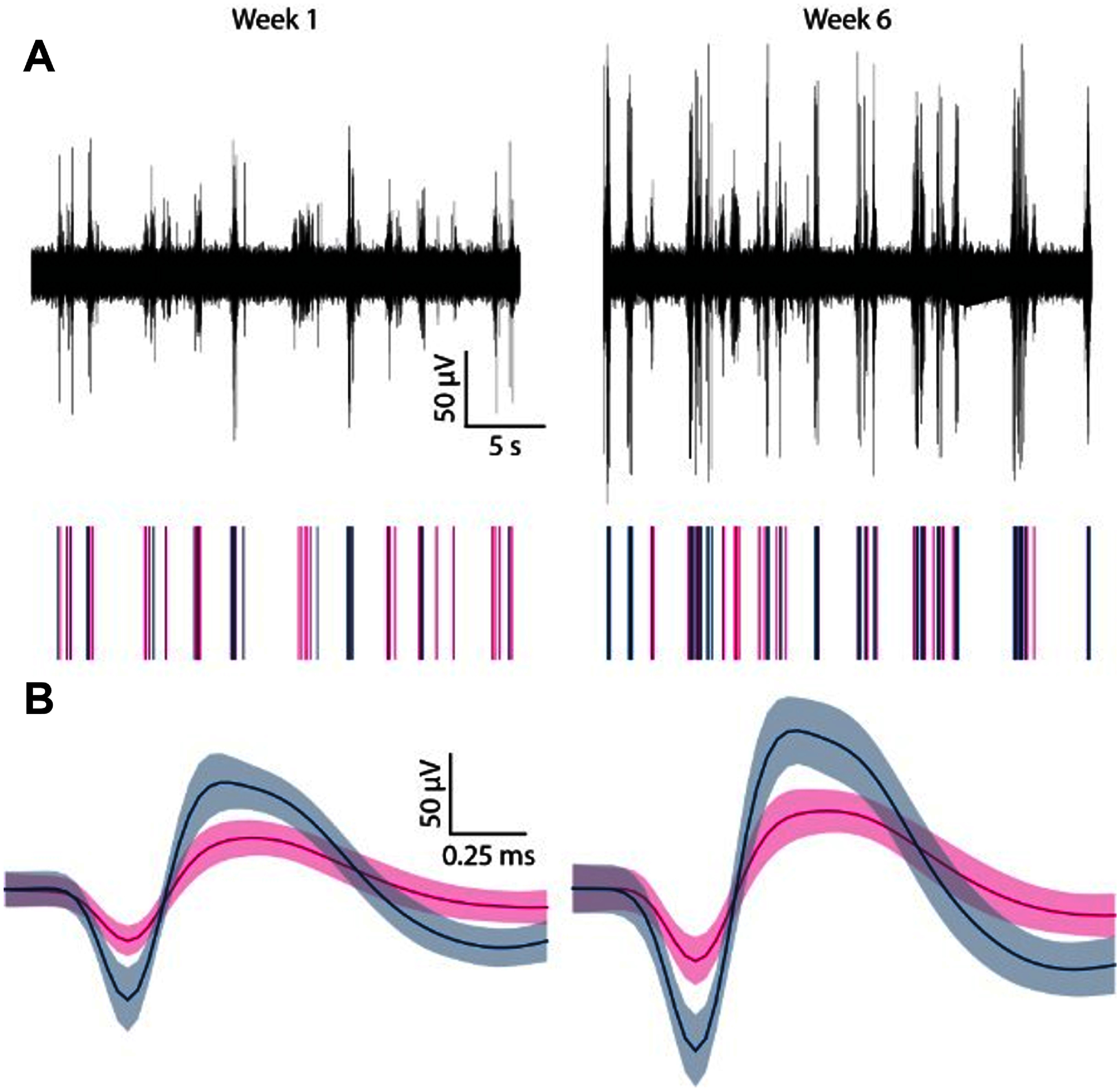
(A) Example of a 30 s segment of band-pass filtered neural activity (300–3000 Hz), with a corresponding raster plot displaying spikes from two representative single units recorded during the epoch at week 1 (left) and week 6 (right). (B) Averaged neuronal waveforms for the identified units, with each color indicating a distinct unit. Solid lines represent the average waveforms and shaded areas show the full range of waveform variability for each unit.

**Figure 6.**
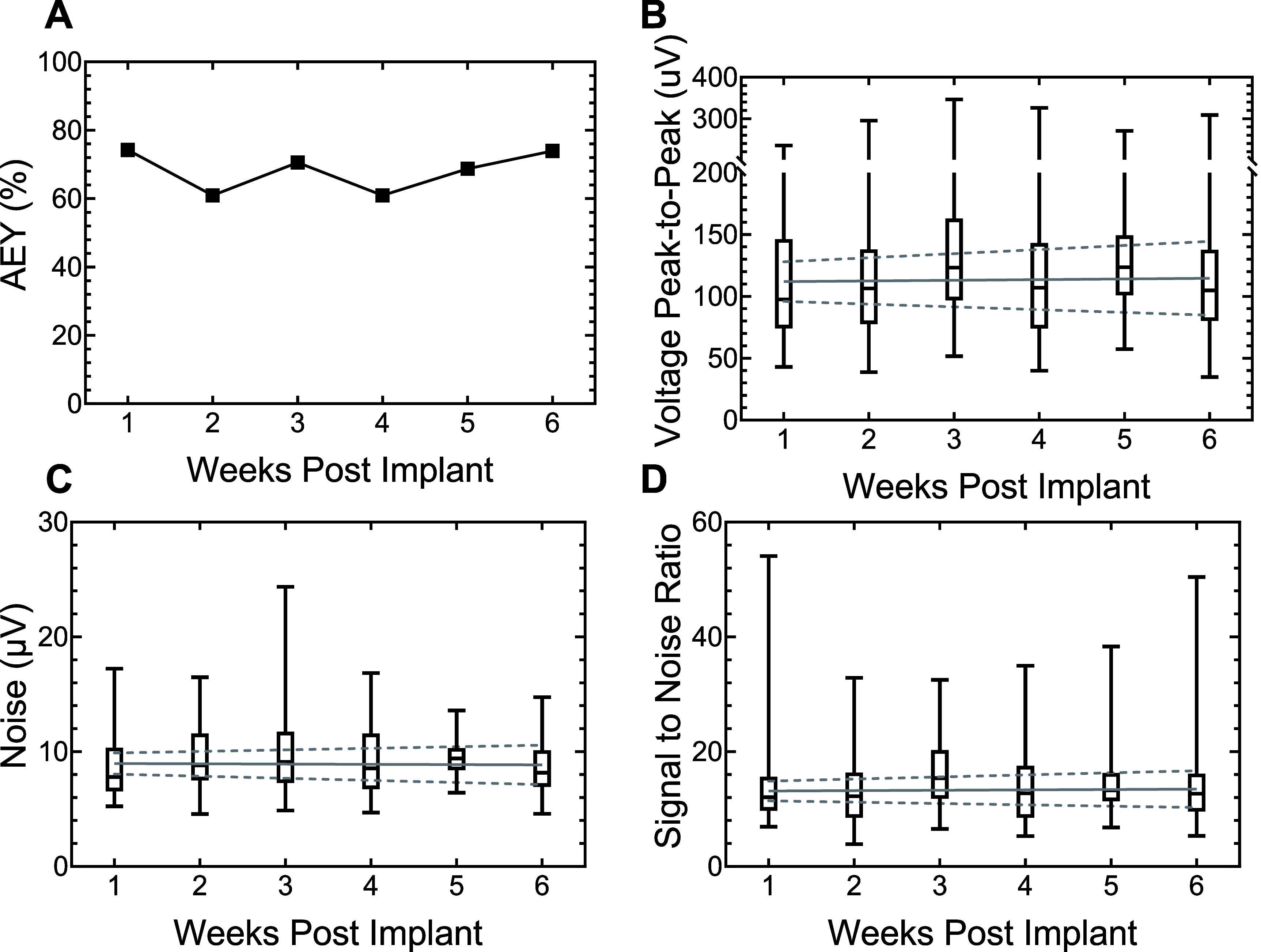
Summary of weekly neural recording data for all functional electrodes across all arrays from weeks 1–6: (A) active electrode yield percentage, (B) peak-to-peak amplitude, (C) RMS noise, and (D) signal-to-noise ratio. Box plots illustrate the 1st and 3rd quartiles, with the median depicted as a horizontal line inside each box, and whiskers representing the range of values from minimum to maximum. The robust linear regression is represented by a solid line with a 95% confidence interval as dashed lines.

Single-unit action potential recordings using RuO*_x_* microelectrodes remained stable over a 6 week period. Bandpass (300–3000 Hz) filtered neural recordings are shown in figure 5(A) for weeks 1 and 6 with corresponding raster plots displaying signals exceeding the −4*σ* threshold. Associated averaged neuronal waveforms appear in figure 5(B). The AEY, calculated across all functional electrodes in all rats throughout the 6 week period, is shown in figure 6(A), with 60%–80% of functional electrodes recording single-unit activity on any given day. Neural recordings from week 0, which correspond to recordings taken on the day of surgery, were excluded from the analysis due to potential effects from surgical anesthesia and noise from surgical equipment. At 1 week post-implantation, 74% of the 112 available electrode channels recorded single-unit activity; at six weeks post-implantation, the AEY percentage remained at 74%, though the number of available channels decreased to 92, as confirmed by electrochemistry.

Regarding the median *V*_pp_, noise levels, and SNR, no significant slope deviation from 0 was observed in robust regression analysis. Median and quartile *V*_pp_ values, noise levels, and SNR, along with robust regression trendlines, are shown in figures 6(B)–(D), respectively. Median and quartile *V*_pp_ values at week 1 were 97.6 *µ*V (75.7 *µ*V, 148 *µ*V), which remained consistent with a slight increase to 105 *µ*V (82.9 *µ*V, 141 *µ*V) by week 6. Noise floor values were similarly stable, measuring 7.8 *µ*V (6.6 *µ*V, 10 *µ*V) at week 1 and 8.1 *µ*V (7.0 *µ*V, 10 *µ*V) at week 6. Consequently, SNR remained largely unchanged, with a median and quartile SNR of 12 (9.8, 15) at week 1 and 12 (9.6, 16) at week 6. No change was observed in the median *V*_pp_ slope (*p* = 0.707, slope = 0.529 *µ*V week^−1^, 95% CI = (−2.24 *µ*V week^−1^, 3.31 *µ*V week^−1^)), and RMS noise values remained stable, with the trend line slope showing no statistical significance from zero (*p* = 0.775, slope = −0.023 *µ*V week^−1^, 95% CI = (−0.18 *µ*V week^−1^, 0.14 *µ*V week^−1^)). Similarly, the SNR showed no change (*p* = 0.671, slope = 0.064 week^−1^, 95% CI = (−0.23, 0.36)). These results demonstrate that RuO*_x_* electrode sites reliably record single-unit action potentials over 6 weeks in the rat cortex.

## 4. Discussion

In this work, we investigated the feasibility of using sputtered RuO*_x_* thin films as low impedance coatings for recording electrodes in rat motor cortex over short chronic implantation periods. Similar to other electrode materials, there is a notable increase in impedance and decrease in CSC_c_ between physiological saline (mISF) and after implantation in cortex [7, 9, 13, 22, 23]. These changes are driven by the higher tissue resistivity compared with mISF and possible adsorption of biomolecules onto the electrode coating [24]. After implantation, the RuO*_x_* impedance and CSC_c_ remained relatively stable over six weeks, with the only major observed change being a mid-frequency (500–5000 Hz) decline in impedance, typified by the 1 kHz impedance shown in figure 3(B). The mean decline in 1 kHz impedance over all electrodes was −0.08 MΩ week^−1^, although progressive tissue encapsulation might be expected to increase impedance, as reported in [7, 9], the *in vivo* mid-frequency impedance of RuO_x_ electrodes in this study was observed to decline. Notably, we observed no increase in the 50 mV s^−1^ CSC_c_ and a very slight decline in 1 Hz impedance over six weeks, suggesting that current leakage under encapsulation or within the implanted connector housing is not creating a low-impedance pathway [5]. However, we did observe a slight increase in the 50 000 mV s^−1^ CV magnitudes, despite the CSC_c_ values not indicating a significant positive trend over time. Whereas the mechanism behind the decline in mid-frequency impedance, also observed in [25], has not been elucidated, ongoing *in vivo* hydration of the RuO*_x_* film is suspected but not confirmed. Additionally, there was also no evidence of RuO*_x_* degradation or delamination as indicated by stable impedance magnitudes at 1 Hz and 30 kHz and CSC_c_, at both 50 mV s^−1^ and 50 000 mV s^−1^, over the six-week implantation period. These results are similar to those reported for the stability of iridium oxide films in similar animal studies [13, 15, 26]. Previous studies have established the stability of the a-SiC, as both encapsulation and as a substrate for MEA fabrication, and no intrinsic changes in the a-SiC are expected to contribute to the observed decrease in 1 kHz impedance [27–29].

It is interesting to note that whereas RuO_x_ is stable in rat cortex, there is a marked difference in 50 mV s^−1^ CSC_c_ values when compared to SIROF electrodes with the same geometric surface area and on the same substrate. SIROF electrodes in rat cortex were observed to have a median CSC_c_ between 50 and 60 mC cm^−2^ in a chronic implantation study [13], while RuO*_x_* electrodes in this study was generally found to be between 25 and 35 mC cm^−2^ while implanted. This may be due to differences in film thickness: the SIROF electrodes had a thickness of approximately 250 nm [13] whereas the RuO_x_ films measured in this study were 120 nm thick. Examining CSC_c_ normalized to film thickness reveals a comparable *in vivo* volumetric CSC_c_ value for both films, where the value of SIROF was 0.2–0.24 mC cm^−2^·nm and RuO*_x_* was 0.21–0.29 mC cm^−2^·nm. These similar volumetric CSC_c_ values of RuO_x_ and SIROF electrode coatings suggest a similar performance in cortex. Given this similarity to SIROF in electrochemical characteristics *in vivo*, recording performance over 6 weeks in rat motor cortex, and amenability to thin film fabrication techniques, as well as reduced cost to fabricate, we believe that RuO*_x_* warrants further investigation as a possible electrode coating for neural interfaces.

The recording performance of the RuO*_x_* electrodes remained consistent over a 6 week implantation time period in rat cortex. As the robust linear regression indicates, there was little change in both the *V*_pp_ and the SNR of these recordings. These data suggest that RuO*_x_* is capable of recording single unit extracellular action potentials for at least a subchronic implantation period of 6 weeks, expanding on the acute implantation work [16]. When compared to microelectrode devices with SIROF, the recording performance of RuO*_x_* electrodes appears comparable, with SIROF and RuO*_x_* both achieving about 75% AEY and a median *V*_pp_ of approximately 100 *µ*V after 6 weeks in rat cortex [13]. Additionally, the *V*_pp_ and SNR data that we report here are similar to PEDOT:PSS electrodes in rat motor cortex, which had a comparable 169 *µ*m^2^ geometric surface area [30].

A limitation of the present study is the small number of animals involved (*n* = 7) and the assignment of causal effects driving changes in electrode performance should be made with caution. Future work will examine the long-term chronic performance of RuO*_x_* electrodes for both neural recording and electrical stimulation.

## 5. Conclusion

In this study, we examined the *in vivo* stability of RuO*_x_* electrodes through electrochemical assessment and neural recordings in a 6 week study in rat motor cortex. We demonstrated that the RuO*_x_* electrodes have an appropriate impedance for single unit recordings and this impedance is maintained over the implantation period. Likewise, the RuO*_x_* CSC_c_, determined from 50 mV s^−1^ and 50 000 mV s^−1^ CVs, was unchanged over six weeks. These electrodes were also able to record from a majority (∼75%) of functional electrodes over the 6 week study and showed no decline in *V*_pp_ or SNR. Overall, these results suggest that RuO*_x_* electrode coatings can provide a stable low-impedance interface for neural recordings over at least a 6 week implantation period. More work remains to evaluate the effectiveness of RuO*_x_* for longer chronic recording performance. Additionally, neural stimulation utilizing RuO*_x_* electrodes remains an interesting avenue for investigation.

## Data Availability

The data cannot be made publicly available upon publication because the cost of preparing, depositing and hosting the data would be prohibitive within the terms of this research project. The data that support the findings of this study are available upon reasonable request from the authors.

## Institutional Animal Care and Use Committee Statement

The study was conducted according to the guidelines approved by the Institutional Animal Care and Use Committee of The University of Texas at Dallas (Protocol 18-13 approved 26 September 2018).
